# Generation of *Leishmania* Hybrids by Whole Genomic DNA Transformation

**DOI:** 10.1371/journal.pntd.0001817

**Published:** 2012-09-20

**Authors:** Adriano C. Coelho, Philippe Leprohon, Marc Ouellette

**Affiliations:** Centre de Recherche en Infectiologie, Université Laval, Québec, Canada; University of Pittsburgh, United States of America

## Abstract

Genetic exchange is a powerful tool to study gene function in microorganisms. Here, we tested the feasibility of generating *Leishmania* hybrids by electroporating genomic DNA of donor cells into recipient *Leishmania* parasites. The donor DNA was marked with a drug resistance marker facilitating the selection of DNA transfer into the recipient cells. The transferred DNA was integrated exclusively at homologous locus and was as large as 45 kb. The independent generation of *L. infantum* hybrids with *L. major* sequences was possible for several chromosomal regions. Interfering with the mismatch repair machinery by inactivating the *MSH2* gene enabled an increased efficiency of recombination between divergent sequences, hence favouring the selection of hybrids between species. Hybrids were shown to acquire the phenotype derived from the donor cells, as demonstrated for the transfer of drug resistance genes from *L. major* into *L. infantum*. The described method is a first step allowing the generation of *in vitro* hybrids for testing gene functions in a natural genomic context in the parasite *Leishmania*.

## Author Summary

*Leishmania* spp. are pathogenic protozoa characterized by a substantial diversity in pathogenesis and virulence despite their considerable synteny at the genome level. The existence of genetic exchange was recently proven experimentally in the sand fly vector where hybrid parasites were isolated and generated. Here, we show the feasibility of generating *Leishmania* hybrids by electroporating genomic DNA of donor cells into recipient *Leishmania* parasites. This methodology was made possible by introducing a drug resistance marker in the donor *Leishmania* cells that could be used for selecting recombinant recipient parasites. Integrations of exogenous DNA fragments as large as 45 kb were possible for several chromosomal regions and took place at homologous loci in recipient *Leishmania* strains. Our observations are the first step for the generation of in vitro hybrids for assessing gene function under natural genomic contexts and this technology may be applicable to other pathogens.

## Introduction

Leishmaniasis is a complex of diseases caused by parasites of the genus *Leishmania* for which there is an estimated 12 million people infected in tropical and subtropical areas of the world [1]. The clinical manifestations of the disease are often correlated with the infecting *Leishmania* species and range from self-healing cutaneous sores (cutaneous leishmaniasis) to deadly visceral pathologies (visceral leishmaniasis) [1]. The *Leishmania* genomes are characterized by a high degree of gene synteny and contain a surprisingly low number of species-specific genes relative to clinical diversity [2], [3]. While *Leishmania* parasites are usually considered as diploid, recent evidence revealed a sizable variation in chromosome copy numbers between species [3], [4]. Hence, the tropism of leishmaniasis is likely to come from a combination of species-specific genes identified to date [2], [5] and from differences in gene copy number and expression between species [6], [7].

The occurrence of a sexual cycle for *Leishmania* has long been debated but the abundance of hybrid parasites described in nature [8]–[10] suggested that the exchange of genetic material can occur in the field. These observations received experimental confirmations where genetic crosses were made between *Leishmania* strains in the sand fly vector [11]–[13]. While powerful, achieving the generation of hybrid parasites *in vitro* would bring a distinct advantage for studying the contribution of genetic loci to the expression of particular phenotypic traits. Since its inception in the early 90's, gene transformation by electroporation has changed the field of parasitology, enabling constant progress in reverse genetic tools. However, these tools allow functional studies mostly at the level of single genes, at least in the natural genomic context [14]. Also, highly homologous sequences are required between the donor and recipient DNAs for successful recombination in *Leishmania* [15], which prevent, in general, the use of single constructs for inactivating the same genes in different species. Cross-species gene replacement was nonetheless described once in *L. donovani* [16]. Given the frequency of hybrid parasites in the field and their importance in shaping *Leishmania* population's heterogeneity, we assessed the possibility of generating cross-species recombinants *in vitro* by heterologous genomic DNA (gDNA) transfection. We describe a knock-in protocol based on whole genome transformation (WGT) that will be useful for studying the role of species-specific genomic loci and of nucleotide polymorphisms pertaining to the biology of *Leishmania*. We show that genomic regions up to 45 kb can be selectively transferred between *Leishmania* species and that inactivating the mismatch repair gene *MSH2* can further facilitate the recovery of cross-species hybrids.

## Methods

### Cell lines, culture conditions and transfections

*L. major* Friedlin, *L. infantum* JPCM5 and *L. infantum* (MHOM/MA/67/ITMAP-263) promastigotes were grown at 25°C in SDM-79 medium supplemented with 10% heat inactivated fetal bovine serum and 10 µg/ml hemin. Transfectants were selected with 300 µg/ml of hygromycin (HYG); 1,500 µg/ml of paromomycin (PM); 100 µg/ml of blasticidin (BLA); 120 µg/ml of puromycin (PUR); or 40 µg/ml of neomycin (NEO). Electroporation was done in 2-mm cuvette using 400 µl of cells (1×10^8^ of parasites) resuspended in HEPES-NaCl buffer [17] at 500 µF, 450 V (2.25 kV/cm) as previously described [18]. Transfection efficiency was evaluated by transfecting 1×10^8^ parasites with 20 µg of gDNA or 5 µg of linear digested DNA, taking into account the number of colonies obtained and the number of parasites transfected. Following electroporation, parasites were grown in drug-free media overnight and then plated on SDM-agar (1% Noble Agar, Nunc.) containing the appropriate drug using the same concentration as in liquid SDM-79 medium. Colonies were counted from SDM-agar plates after growing 10 to 14 days at 25°C and the transformation efficiencies expressed per 10 µg of DNA.

The *L. major* Friedlin MF80.3 mutant was selected from a cloned parental population using a stepwise selection until they were resistant to 80 µM of miltefosine (MF) [19]. Miltefosine (MF) was purchased from Cayman Chemical (Ann Harbor, USA) and trivalent antimony (SbIII) was purchased from Sigma (Saint Louis, USA). N-methyl-N′-nitro-N-nitrosoniguanidine (MNNG) was purchased from Sigma (Saint Louis, USA) and was dissolved in 100% DMSO. Growth curves were obtained by measuring absorbance at 600 nm as previously described [20].

### DNA manipulations

Total DNA was isolated using DNAzol reagent (Invitrogen) as recommended by the manufacturer. For quantitative Southern blots, the genomic DNA was digested with appropriate restriction enzymes and migrated in 0.8% agarose gels. Southern blots, hybridizations and washes were performed following standard protocols [21].

For every gene inactivation cassette, two pairs of primers were used to amplify regions of 500–600 bp upstream and downstream of the target gene. These DNAs were then fused to the *NEO*, *HYG* or *BLA* genes by overlap extension PCR [22], [23]. After electroporation, the integration of the inactivation cassette was confirmed by PCR and Southern blots. The inactivation of the gene *pyridoxal kinase* (*LmjF30.1250*) in *L. major* was previously described [19]. The pSP72-αBLAα-*LRP* plasmid was generated by cloning the *Leucine Rich Repeat Protein* (*LRP*) gene amplified from *L. major* with primer F-LRP (5′ GCG **GATATC**GCTGTTGGTGTTCGTGTCGTC) and Primer R-LRP (5′ GCG **ATCGAT**CAGAGGCGGAGTGGGCTGTCC) into *Eco*RV and *Cla*I restriction sites of the pSP72-αBLAα- vector. The pSP-αPURα-*MSH2* plasmid was generated by cloning the *MSH2* gene amplified from *L. infantum* with primer F-MSH2 (5′ CGC**TCTAGA**CGCACATGCACCTACGCACG) and Primer R-MSH2 (5′ CGC**TCTAGA**CAAACAAGGATAGCGAGAAG) into the single *Xba*I restriction site of the psp72-αPURα vector. All the primers used for these constructs are listed in Table S1.

The pulse field gel electrophoresis was performed as previously described [24]. Briefly, we prepared low-melting point agarose blocks containing *Leishmania* parasites resuspended in HEPES buffer at a cell density of 1×10^8^ parasites/ml. Parasites were lysed by incubating the blocks in lysis buffer (0.5 M EDTA [pH 9.5]; 1% SLS; 50 mg/ml of proteinase K). Chromosomes were resolved by a BioRad (Hercules, California, USA) contour-clamped homogeneous electric field (CHEF) mapper at a constant temperature of 14°C. *Saccharomyces cerevisiae* chromosomes were used as molecular weight markers. Chromosomes were revealed by ethidium bromide staining.

### DNA amplification and sequencing

Multilocus sequencing analyses were performed with *Leishmania* hybrids by amplifying the genes located in the vicinity of the integrated selection marker using primers specific for genomic regions conserved between *L. major* and *L. infantum*. Primers were designed with Primer3 [25] and are listed in Table S2. PCR products were amplified using Phusion High Fidelity DNA polymerase (New England Biolabs, Inc.) and sequenced by conventional Sanger sequencing. Sequences were analyzed with the Lasergene software (DNASTAR, Inc.) and compared to the *L. major* and *L. infantum* sequences available at GeneDB (www.genedb.org).

### Whole genome sequencing and analysis

Genomic DNAs were prepared from mid-log phase cultures of *L. infantum* 263 transfected with gDNA derived from *L. major* MF80.3ΔLmjF13.1540::NEO/LmjF13.1540 and from the clone 1 of *L. infantum* JPCM5 transfected with gDNA derived from *L. major* LmΔ*LRP*::NEO/LRP as previously described [26]. Their sequences were determined by Illumina HiSeq1000 101-nucleotides paired-end reads which assembled into 4849 and 2774 contigs of at least 500 nucleotides for the *L. infantum* 263 and *L. infantum* JPCM5 hybrids, respectively. Sequence reads from each hybrid were aligned to the reference genome *L. infantum* JPCM5 [2] available at TriTrypDB (version 4.0) [27] using the software bwa (bwa aln, version 0.5.9) with default parameters [28]. The maximum number of mismatches was 4, the seed length was 32 and 2 mismatches were allowed within the seed. The detection of single nucleotide polymorphisms (SNPs) was performed using samtools (version 0.1.18), bcftools (distributed with samtools) and vcfutils.pl (distributed with samtools) [29], with a minimum of three reads to call a potential variation prior to further analysis. The sequence data are available at the EMBL European Nucleotide Archive (http://www.ebi.ac.uk/ena), accession number ERP001431 (samples ERS138995 and ERS138996 corresponding to the *L. infantum* 263 hybrid transfected with gDNA derived from LmMF80.3ΔLmjF13.1540::NEO/LmjF13.1540 and the *L. infantum* JPCM5 hybrid transfected with LmΔ*LRP*::NEO/LRP gDNA, respectively). Several python (version 2.4.3) scripts and bash (version 3.2) scripts were created to further analyze the data. The quality assessment software samstat (v1.08) was used to generate quality reports [30].

## Results

### Donor line generations

**Figure 1:**
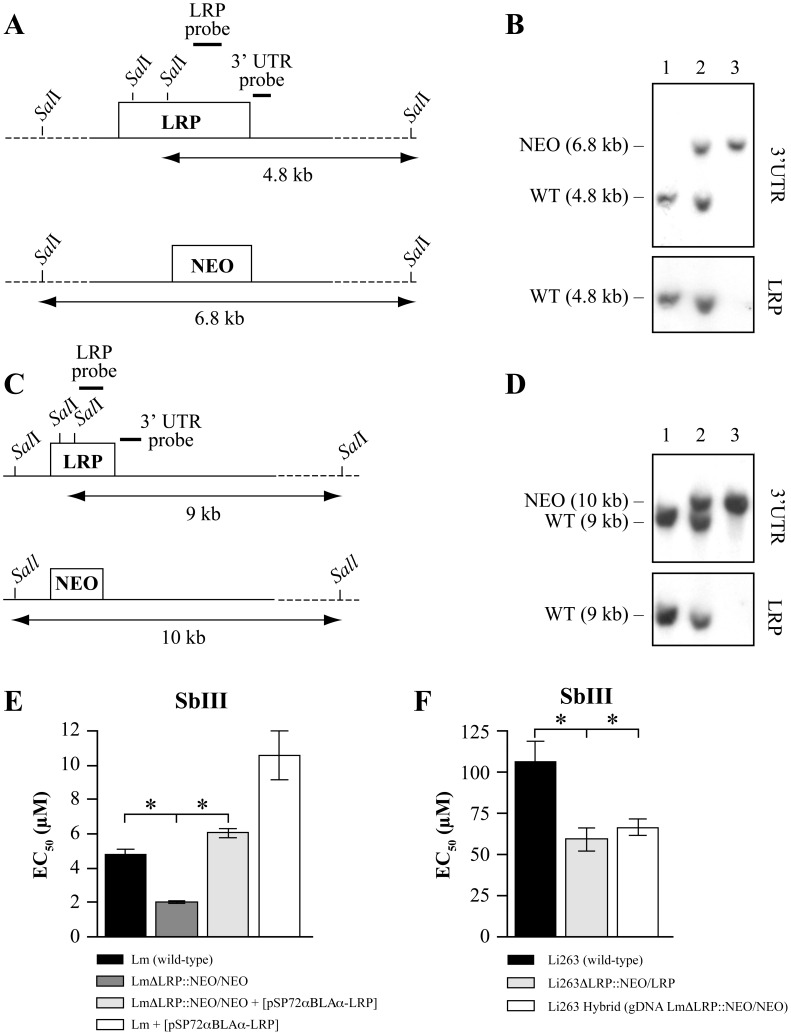
Targeted replacement of the *L. major* and *L. infantum LRP* gene. Schematic drawing of the *LRP* locus with *Sal*I sites of *L. major* and *L. infantum* (A, C). (B) Southern blot analysis of *L. major* Friedlin genomic DNA digested with *Sal*I and hybridized with a 3′ UTR probe of *LRP* gene (a ∼500 bp fragment just downstream the stop codon of *LRP* gene) (upper panel) and then with a LRP PCR amplified gene fragment of ∼500 bp (bottom panel). (D) Southern blot analysis of *L. infantum* 263 genomic DNA digested with *Sal*I and hybridized with a 3′ UTR probe of *LRP* gene (a ∼500 bp fragment just downstream the stop codon of *LRP* gene) (upper panel) and then with the LRP PCR specific fragment (bottom panel). Molecular weight is indicated on the left. Lane 1, *Leishmania* WT cells; Lane 2, *Leishmania* SKO cells (LRP/NEO); 3, *Leishmania* DKO cells (NEO/NEO) at the *LRP* locus obtained by loss of heterozygosity. (E, F) SbIII susceptibilities of *L. major* and *L. infantum LRP* mutants and hybrid parasites. The EC_50_ values were determined by culturing promastigotes parasites in the presence of increasing concentrations of SbIII. The average of three independent experiments is shown with a statistical significance observed by Student's t-test (p<0.05) (*).

Our work was carried out with *L. major* and *L. infantum*, two species respectively responsible for cutaneous and visceral leishmaniasis that have an estimated 20–100 million years of divergence [31], [32]. Our strategy for selecting hybrid parasites was to introduce selectable markers (usually the *NEO* gene) into specific *L. major* genes, to electroporate total gDNA extracted from these recombinant parasites into recipient cells and to recover recombinant hybrids thriving under selection pressure. The first locus that was studied encodes for a leucine rich repeat protein (LRP) on chromosome 34 (LmjF34.0550) that we showed to be involved in resistance to antimonials [33], the chemotherapeutic mainstay against *Leishmania*. We generated mutants haploid for *LRP* by inserting *NEO* cassettes made to target either the *LRP* gene of *L. major* Friedlin (LmjF34.0550) or *L. infantum* strain 263 (LinJ34_V3.0570) (Figure 1), giving rise to the LmΔ*LRP*::NEO/LRP and Li263Δ*LRP*::NEO/LRP haploid lines, respectively (Figure 1B, 1D, lanes 2). We also successfully obtained *LRP* null mutants for both species (LmΔ*LRP*::NEO/NEO and Li263Δ*LRP*::NEO/NEO) by loss of heterozygosity [34] after one round of allelic inactivation and higher drug selection (Figure 1B, 1D, lanes 3). Consistent with the role of LRP in antimonial resistance, the LmΔ*LRP*::NEO/NEO and Li263Δ*LRP*::NEO/NEO mutants were more sensitive to antimonials (Figure 1 and data not shown). We also generated haploid mutants for genes present on chromosome 1 (LmjF01.0315), 5 (LmjF05.0610), 13 (LmjF13. 1540) and 30 (LmjF30.1250) of *L. major* Friedlin. With the exception of the *L. major* LmΔ*PK*::NEO/PK mutant haploid for LmjF30.1250 (pyridoxal kinase gene) that has already been published [19], all other single *NEO* disruptions were generated for this study and molecular evidence for the haploid mutants can be found in Figure S1.

### Generation of parasite hybrids

**Figure 2:**
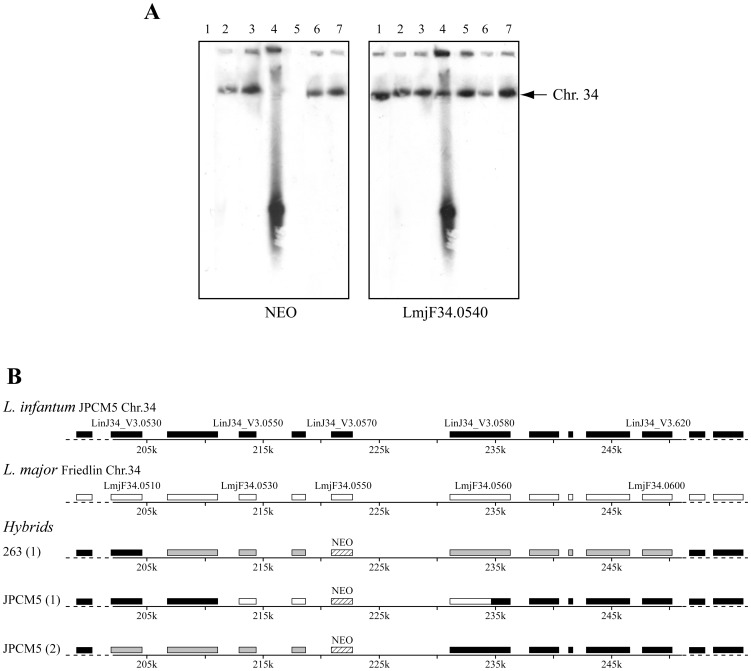
Whole genome transformation in *Leishmania* and cross-species hybrid formation. Genomic DNAs derived from LRP haploid mutants of *L. infantum* 263 (Li263Δ*LRP*::NEO/LRP) or *L. major* Friedlin (LmΔ*LRP*::NEO/LRP) were used to transfect *L. infantum* 263 or *L. infantum* JPCM5 (WT) parasites. (A) Chromosomes were separated by CHEF, transferred, and hybridized with probes specific for the *NEO* gene or for LmjF34.0540 gene located just upstream of the *LRP* gene on chromosome 34. The 2,000 kb chromosome 34 is indicated by an arrow on the right. Lane 1, *L. infantum* 263 WT; Lanes 2–4, *L. infantum* 263 transfected with Li263Δ*LRP*::NEO/LRP gDNA (2); LmΔ*LRP*::NEO/LRP gDNA (3–4); Lane 5, *L. infantum* JPCM5 WT; Lanes 6–7, *L. infantum* JPCM5 transfected with Li263Δ*LRP*::NEO/LRP gDNA or with LmΔ*LRP*::NEO/LRP gDNA, respectively. (B) Schematic map of the genomic region encompassing the *LRP* gene of *L. infantum* JPCM5 (black boxes) and *L. major* Friedlin (white boxes). Hybrids with one allele of *L. major* and one allele of *L. infantum* are shown in gray. The genomic regions exchanged in the hybrids were mapped by sequencing individual amplified genes using primers amplifying both *L. major* and *L. infantum* genes (Tables S3, S4, S5) located in the vicinity of the integrated *NEO* marker. The sequence of the hybrid JPCM5 (1) was also obtained by next generation sequencing. The extent of the genomic DNA exchanged is indicated after sequence analysis. In the JPCM5 clone 1 hybrid, both *L. infantum* alleles were replaced by *L. major*.

Whole genome transformation (WGT) has proven useful when working with naturally transformable microorganisms [35], [36] and electroporation was tested for rendering *Leishmania* cells competent to receive total gDNAs from other *Leishmania* strains. We first electroporated total gDNA derived from *L. infantum* Li263Δ*LRP*::NEO/LRP into wild-type (WT) *L. infantum* 263 recipients cells and recovered recombinant clones resistant to paromomycin, a drug for which *NEO* bestows resistance. The *NEO* marker integrated at the appropriate locus in most clones (Table 1), as determined by Southern blot analysis of digested DNA (data not shown) but also of chromosome sized blots since a *NEO* probe hybridized to chromosome 34 in recombinant clones but not in the initial *L. infantum* 263 WT cells (Figure 2A, lanes 1 and 2). Similar results were obtained for *L. major* Friedlin, where electroporation of gDNA derived from *L. major* LmΔ*LRP*::NEO/LRP single disruptant into *L. major* Friedlin WT parasites led to transfectants growing in the presence of paromomycin (data not shown). Again, the *NEO* gene integrated properly at chromosome 34 in the recovered recombinants, as determined by Southern blot analysis of chromosome sized blots (data not shown). We also succeeded in electroporating total gDNA derived from *L. infantum* Li263Δ*LRP*::NEO/LRP into *L. infantum* JPCM5 WT recipients and isolated recombinants which have integrated the *NEO* marker at the level of chromosome 34 (Figure 2A, compare lanes 5 and 6). The latter results indicated that gDNAs electroporated in *Leishmania* can integrate at homologous loci, at least in the same strains or species.

**Table 1:** Summary of generation of *Leishmania* hybrids using different gDNA.

Recipient *Leishmania* strain	gDNA donor *Leishmania*	Transfection efficiency	% of stable and episomal transformants
*L. major* Friedlin	LmΔ*LRP*::NEO/LRP	2×10^−8^	95 and 5%
*L. infantum* JPCM5	LmΔ*LRP*::NEO/LRP	1.2×10^−8^	83 and 17%
*L. infantum* 263	LmΔ*LRP*::NEO/LRP	2.4×10^−8^	90 and 10%
*L. infantum* JPCM5	Li263Δ*LRP*::NEO/LRP	10^−8^	100 and 0%
*L. infantum* 263	Li263Δ*LRP*::NEO/LRP	2.2×10^−8^	90 and 10%
*L. infantum* Li263Δ*MSH2*::HYG:BLA	LmΔ*LRP*::NEO/LRP	8×10^−8^	83 and 17%
*L. infantum* JPCM5	LmΔ*PK*::NEO/PK	1.5×10^−8^	83 and 17%
*L. infantum* 263	LmΔ*PK*::NEO/PK	2.3×10^−8^	70 and 30%
*L. infantum* Li263Δ*MSH2*::HYG:BLA	LmΔ*PK*::NEO/PK	7×10^−8^	80 and 20%
*L. infantum* 263	LmΔLmjF05.0610::NEO/LmjF05.0610	0	0
*L. infantum* Li263Δ*MSH2*::HYG:BLA	LmΔLmjF05.0610::NEO/LmjF05.0610	4.6×10^−8^	100 and 0%
*L. infantum* 263	LmMF80.3ΔLmjF*13.1540*::NEO/LmjF13.1540	4×10^−8^	10 and 90%

Transfection efficiency was calculated by dividing the number of colonies by the number of transfected cells. The relative abundance of stable and episomal transformants were determined on clones by CHEF, followed by Southern blot as described in the Methods using the respective drug resistance marker as probe.

We then tested whether we could cross the species barrier and electroporated total gDNA derived from *L. major* LmΔ*LRP*::NEO/LRP into either *L. infantum* 263 or JPCM5 WT cells. This indeed seemed to have occurred since a *NEO* marker was found to have integrated at the proper chromosome in both *L. infantum* strains (Figure 2A, lanes 3 and 7). This is significant as the construct used for inactivating the targeted allele is species-specific but the use of gDNAs allowed crossing the species barrier. The integration of the *NEO* marker into the homologous locus did not occur in all transfectant clones however, as the smear obtained in PFGE blots for one *L. infantum* 263 transfectant suggested a circularization of a *NEO* containing DNA fragments (Figure 2A, lane 4). This was indeed confirmed by the recovery of circular episomes from this transfectant by alkaline lysis (data not shown). In most transformation experiments the majority of clones had donor DNA integrated but episomal DNA was also observed in other clones (Table 1). While *L. major* and *L. infantum* are highly syntenic [2], there are nonetheless differences between homologous genes at the nucleotide level (mean 90% genome identity) and we capitalized on these natural single nucleotide polymorphisms (SNPs) to measure the extent of exchanged DNA between both species. By targeted sequencing of genomic regions located upstream and downstream of the *NEO* gene in one hybrid clone of *L. infantum* 263 and two hybrid clones of *L. infantum* JPCM5, we could infer that DNA fragments ranging from 20 kb to 40 kb were exchanged at the *LRP* locus between *L. major* and *L. infantum* (Figure 2B). For example, when we sequenced the *LmjF34.0540* gene (*LinJ34_V3.0560*) in the 263 hybrid clone 1 we found an hybrid sequence with one allele with *L. major* and one allele with *L. infantum* sequences (Table S3). Sequencing of PCR fragments was done similarly with genes upstream and downstream of *LinJ34_V3.0560* to find the first genes (*LinJ34_V3.0530* and *LinJ34_V3.0630*) upstream and downstream *NEO* with exclusively *L. infantum* sequences. The genes in between were hybrids between *L. infantum* and *L. major* (Table S3). In this specific hybrid transfectant, the contiguous DNA transferred was estimated to be at least 40 kb. We used a similar strategy of multilocus sequencing to characterize the extent of DNA exchange in the hybrids JPCM5 clone 1 (Table S4) and clone 2 (Table S5). Interestingly, both *L. infantum* alleles were replaced by the *L. major* sequences in the JPCM5 clone 1 hybrid parasite, most probably by loss of heterozigocity (Figure 2B and Table S4). We also performed paired-ends next generation sequencing of the whole genome of the *L. infantum* JPCM5 hybrid clone 1. Sequence analysis revealed a single stretch of 1055 SNPs (381 within coding sequences) derived from *L. major* that spanned positions 212,950 to 234,918 (48 SNPs/kb). In contrast, only 131 SNPs were detected for the rest of the 1.8 Mb sequence of chromosome 34 upstream and downstream of the integrated *L. major* DNA fragment (0.07 SNP/kb). Most importantly, analysis of the sequenced genome revealed that no other genomic fragments derived from *L. major* Friedlin were co-transferred elsewhere in the genome (Figure 2B, hybrid JPCM5 (1)). Thus, targeted sequencing confirmed that we could generate *L. infantum* hybrid parasites containing up to 40 kb of contiguous *L. major* Friedlin sequence and whole genome sequencing confirmed this and also informed that no other DNAs inserted elsewhere in the genome. The integration was stable and maintained in the absence of selective pressure (not shown). Importantly, hybrids acquired the phenotype of the donor cells since every *L. infantum* 263 or *L. infantum* JPCM5 hybrid clones with a *LRP/NEO* integrated DNA had an increased sensitivity to SbIII (Figure 1F and data not shown).

**Figure 3:**
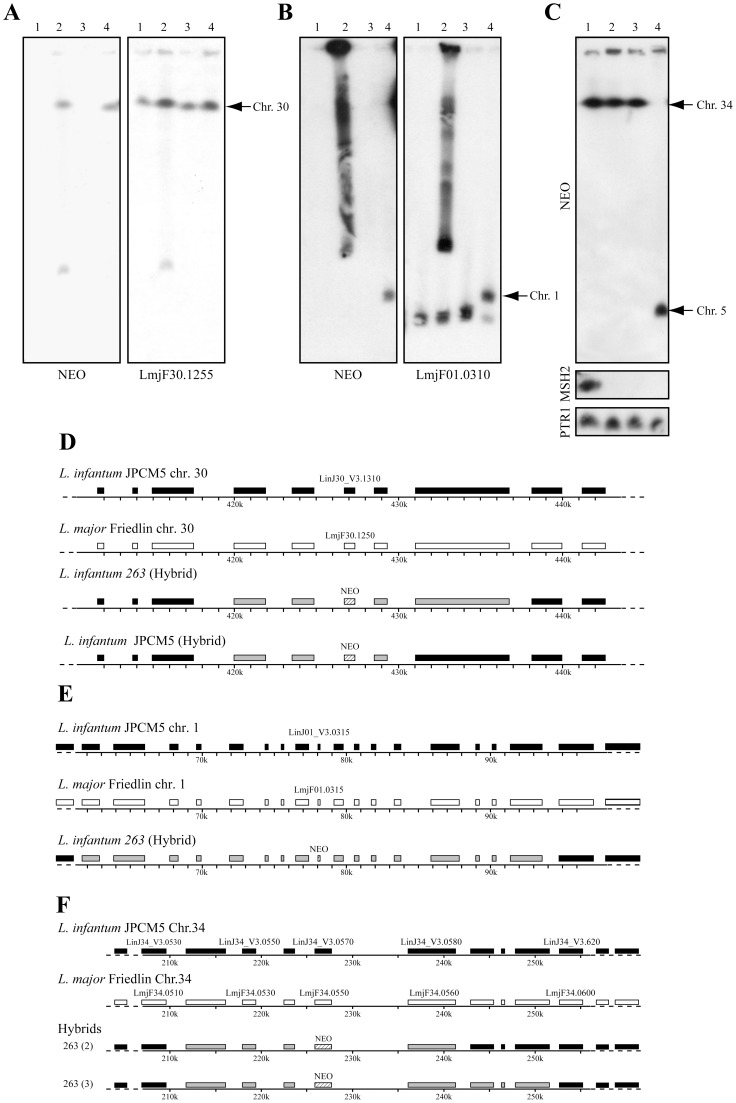
Generation of *L. infantum* hybrids. Genomic DNAs derived from *L. major* Friedlin *NEO* recombinants were electroporated into *L. infantum*. Chromosomes of putative hybrids were separated by pulsed-field gel electrophoresis. (A) *L. infantum* 263 and JPCM5 wild-type cells (lanes 1, 3) were transformed with gDNA derived from LmΔ*PK*::NEO/PK (LmjF30.1250) (lanes 2, 4). The hybridization of chromosome sized blots with probes derived from the *NEO* (left panel) or LmjF30.1255 (right panel) genes confirmed the proper integration of *L. major* DNA in *L. infantum PK* locus. (B) *L. infantum* JPCM5 and 263 WT cells (lanes 1, 3) were transfected with LmΔLmjF*01.0315*::NEO/LmjF01.0315 gDNA (lanes 2, 4). The hybridization of chromosome sized blots with probes derived from the *NEO* (left panel) or LmjF01.0310 (right panel) genes confirmed the proper integration of *L. major* DNA in *L. infantum.* (C) Generation of hybrids into *L. infantum* 263 *MSH2* null mutants. The hybridization of chromosome sized blots with probes specific for the *NEO* gene (upper panel) confirmed the proper integration of *L. major*-derived DNA at the level of targeted chromosomes in *L. infantum*. Hybridizing the same blots with a probe specific for *MSH2* (middle panel) confirmed the *MSH2*-null mutant status of the *L. infantum* recipients while *PTR1* hybridization served as a loading control. *L. infantum* 263 wild-type parasites transfected with gDNA derived from *L. major* LmΔ*LRP*::NEO/NEO (lane 1); *L. infantum* Li263Δ*MSH2*::HYG/BLA transfected with gDNA derived from *L. major* LmΔ*LRP*::NEO/NEO (lanes 2 and 3) or *L. major* LmΔLmjF05.0610::NEO/LmjF05.0610 (lane 4). Schematic map of the genomic region encompassing the *PK* (D), the LmjF01.0315/LinJ01_V3.0315 (E), and *LRP* (F) genes of *L. infantum* JPCM5 (black boxes) or *L. major* Friedlin (white boxes) and hybrid regions with one allele derived from *L. major* and one allele from *L. infantum* (grey boxes) as determined by multilocus PCR sequencing (Tables S6, S7, S8, S9, S10).

The ability to select for the integration of *NEO*-marked gDNA from *L. major* into *L. infantum* recipients was not unique to the *LRP* locus. We recently inactivated one allele of the pyridoxal kinase gene in *L. major* (*LmjF30.1250*) [19] and the electroporation of gDNA derived from this haploid mutant (LmΔ*PK*::NEO/PK) into *L. infantum* 263 and JPCM5 WT cells allowed recovering hybrid transfectants thriving under paromomycin pressure. Hybridization of chromosome sized blots confirmed the proper integration at the level of chromosome 30 (Figure 3A) and targeted sequencing of individual genes upstream and downstream of the NEO marker in both *L. infantum* hybrid strains (Tables S6 and S7) estimated that DNA fragments of 12 and 18 kb derived from *L. major* Friedlin had been exchanged at the *PK* locus of both *L. infantum* strains (Figure 3D). In the *L. infantum* 263 recipient, we observed both integration at the level of the chromosomal locus and a circular amplicon (Figure 3A, lane 2).

**Figure 4:**
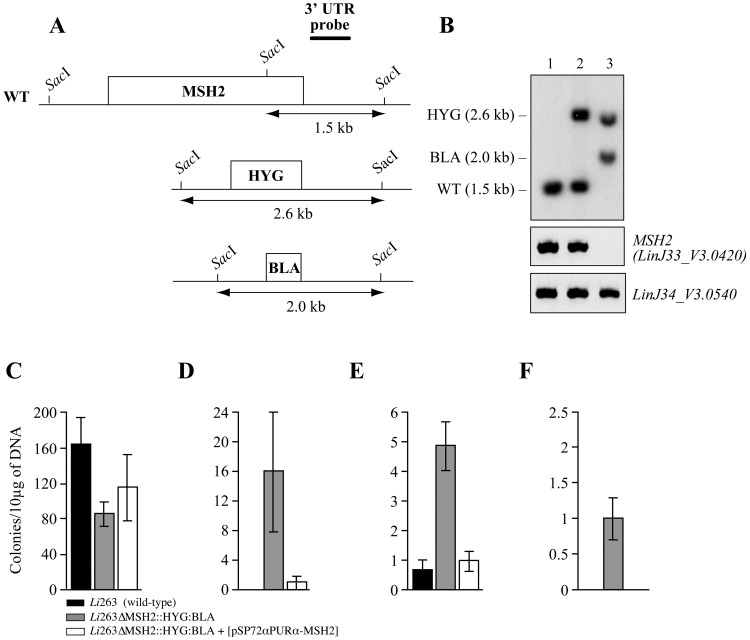
The inactivation of *MSH2* increases the frequency of generation of cross-species hybrids. (A) Schematic map of the wild-type and inactivated *MSH2* alleles of *L*. *infantum*. (B) Southern blot analysis of gDNA derived from *L. infantum* WT (Lane 1), *L. infantum MSH2* haploid mutant (Lane 2) and *L. infantum MSH2* null mutant (Lane 3) digested with *Sac*I and hybridized with a probe specific for the 3′ UTR of *MSH2* (upper panel). The absence of *MSH2* gene was confirmed by PCR using a pair of internal primers of the gene (middle panel); and primers for the amplification of *LinJ34_V3.0540* were used as control (lower panel). Molecular weight is indicated on the left of the blot. (C–F) The number of recombinant clones obtained from 1×10^8^ *L. infantum* 263 WT parasites (black bars), *L. infantum* Li263Δ*MSH2*::HYG:BLA (grey bars) and *L. infantum* Li263Δ*MSH2*::HYG:BLA complemented *in trans* for *MSH2* (white bars) transfected with a linear LinJ28_V3.0330:NEO DNA PCR fragment amplified from *L. infantum* (C), a linear LmjF30.1250:NEO (PK) DNA PCR fragment amplified from *L. major* (D), gDNA derived from *L. major* Friedlin LmΔ*LRP*::NEO/NEO (E), or gDNA derived from *L. major* Friedlin LmΔLmjF05.0610::NEO/LmjF05.0610 (F). The average of at least 3 experiments is indicated and the number of colonies is indicated per 10 µg of DNA.

To test the generality of hybrid formation, we generated haploid *L. major* Friedlin mutants for the LmjF01.0315 (LmΔLmjF01.0315::NEO/LmjF01.0315) or LmjF05.0610 (LmΔLmjF05.0610::NEO/LmjF05.0610) genes (Figure S1), both coding for proteins of unknown function. Electroporating *L. infantum* 263 WT cells with gDNA derived from LmΔ01.0315::NEO/LmjF01.0315 yielded paromomycin-resistant hybrids that had properly integrated the *NEO* gene at the level of chromosome 1 (Figure 3B, lane 4). Using our targeted DNA sequencing approach of individual genes around the NEO marker (Table S8), we could estimate that a 40 kb contiguous *L. major* DNA fragment replaced the *L. infantum* sequence in one allele (Figure 3E). Two bands were observed when hybridizing with the probe specific to chromosome 1 (Figure 3B), but this is probably related to the size polymorphism already reported for this chromosome [37]. It is hence probable that the *NEO* marker integrated in the chromosome with higher molecular weight (Figure 3B, lane 4). The integration of the *NEO* marker did not occur in the chromosome 1 of *L. infantum* JPCM5 however, as the smear obtained in PFGE blots for this transfectant indicated a circularization of the *NEO* cassette (Figure 3B, lane 2). However, the same *L. infantum* cells transfected with gDNA from LmΔLmjF05.0610::NEO/LmjF05.0610 failed to lead to any transfectants (Figure 4F). This suggested that hybrid formation differed between genomic loci targeted by WGT.

### Improving recipient cells

The efficiency of generation of hybrids containing up to 40 kb of sequences from another species was found to be low. Indeed, while the efficiency of transfection for *NEO* disruption PCR constructs targeting single genes was calculated to be 1×10^−7^, this was decreased about 10 fold when transfecting whole gDNA (Table 1). The lack of integration observed for some gDNAs, for example the gDNA derived from *L. major* LmΔLmjF05.0610::NEO/LmjF05.0610 into *L. infantum* (Figure 4F), could be due to several reasons but it could possibly occur if recombination between non identical DNAs was made more permissive. We first assessed whether overexpressing the *RAD-51* recombinase gene (*LinJ28_V3.0580*) in *L. infantum* parasites [38] could increase recombination efficiency and lead more easily to hybrids but this has not been the case (results not shown). We then assessed whether the generation of hybrids with heterologous DNA would be more efficient in cells impaired for the mismatch repair (MMR) machinery since it was showed to prevent recombination between divergent sequences [39], [40]. In the related trypanosomatid parasite *Trypanosoma brucei*, the inactivation of the MMR gene *MSH2* increased the efficiency of recombination between mismatched DNAs [41]. *L. infantum* has a single *MSH2* gene (*LinJ33_V3.0420*) and we generated a *L. infantum* 263 *MSH2* null-mutant (Li263Δ*MSH2*::HYG/BLA) by two successive rounds of allelic inactivation using HYG and BLA inactivation cassettes (Figure 4A) conferring resistance to hygromycin and blasticidin, respectively. The inactivation of *MSH2* in Li263Δ*MSH2*::HYG/BLA was confirmed by Southern blot analysis of restricted DNA and by PCR using specific *MSH2* primers (Figure 4B). A *MSH2* chromosomal null mutant complemented in *trans* for *MSH2* was also generated by transfecting Li263Δ*MSH2*::HYG/BLA with the rescue plasmid pSP72αPURα-*MSH2* (data not shown).

Impairing with the MMR machinery is usually associated with an increased tolerance to N-methyl-N′-nitro-N-nitrosoniguanidine (MNNG), an alkylating agent that interferes with the proper replication of DNA by methylating the O^6^ position of guanine. While the *L. infantum MSH2*-deficient line had no significant growth difference in comparison to wild-type cells, we observed a small but significant increase in resistance to MNNG (Figure S2). Interestingly, the *MSH2* null mutant was more proficient in recombination for small heterologous linear DNA fragments (Figure 4D) but not for those using homologous fragments (Figure 4C). Most importantly, the inactivation of *MSH2* in *L. infantum* also allowed recovering a higher number of recombinants following electroporation with cross-species gDNA derived from *L. major* LmΔ*LRP*::NEO/LRP (Figure 4E). By sequencing PCR fragments for genes upstream and downstream of *NEO* (Tables S9 and S10), we could determine that fragments of 35–45 kb were transferred in two independent clones of the *L. infantum MSH2* null mutant hybrids (Figure 3F). Transfection in the *MSH2* null mutant also allowed recovering recombinants when transfecting with gDNAs that otherwise did not led to hybrids with WT recipients. Indeed, while we could never recover paromomycin-resistant transfectants when transforming *L. infantum* 263 WT parasites with gDNA extracted from a *L. major* Friedlin line inactivated for one allele of the *LmjF05.0610* gene (LmΔLmjF*05.0610*::NEO/LmjF05.0610) (Figure S1), the transformation of the *L. infantum MSH2* null mutants consistently yielded recombinant parasites (Figure 4F). The hybridization of chromosome sized blots confirmed that the *NEO* gene integrated in the proper chromosome for every aforementioned WGTs (Figure 3C).

### Application of whole genome transformation to study the role of a point mutation in drug resistance

**Figure 5:**
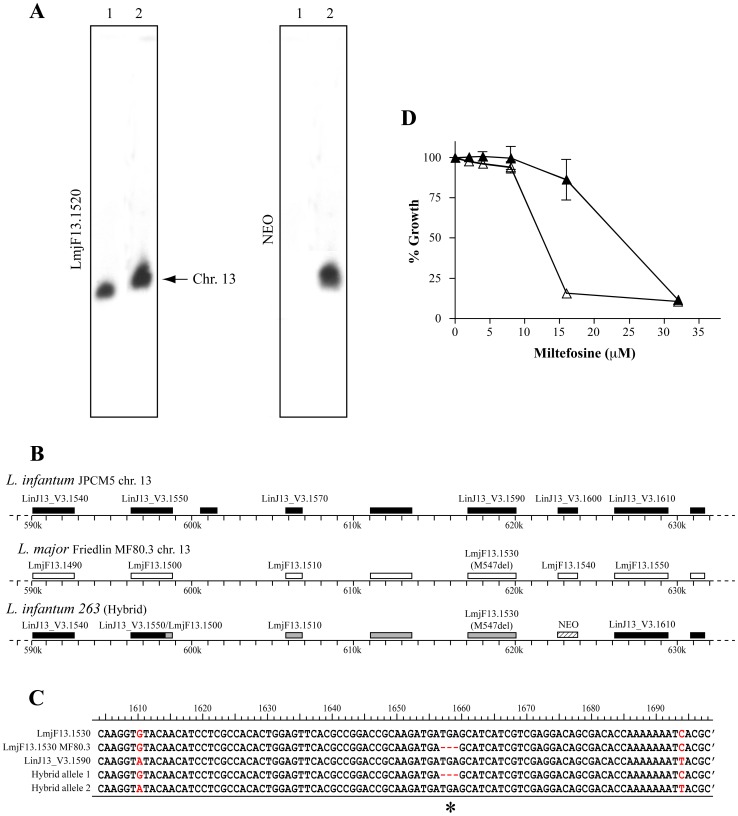
Cross species functional phenotypic transfer. (A) *L. infantum* 263 WT parasites were transfected with total gDNA derived from *L. major* Friedlin LmMF80.3ΔLmjF*13.1540*::NEO/LmjF13.1540 (Figure S1C). Chromosome sized blots were hybridized with probes specific for the *LmjF13.1520* gene (left panel), located just upstream of the *MT* gene on chromosome 13, or for the *NEO* marker (right panel). Lane 1, *L. infantum* 263 WT; Lane 2, *L. infantum* hybrid for *L. major* LmMF80.3 gDNA at its *MT* locus. (B) Schematic map of the genomic region encompassing the *MT* gene of *L. infantum* JPCM5 (black boxes), *L. major* Friedlin MF80.3 (white boxes), and the hybrid region (grey boxes). The genomic regions exchanged in the *L. infantum* 263 hybrid were mapped by sequencing the genes located in the vicinity of the integrated *NEO* marker (Table S11) and by whole genome short reads next generation sequencing. (C) Sequence of the *MT* genes of *L. major* Friedlin (LmjF13.1530), *L. major* Friedlin MF80.3 [19] and *L. infantum* JPCM5 WT (LinJ13_V3.1590) respectively. The *MT* gene from *L. infantum* hybrids was amplified and cloned into the pGEM-T-easy vector. The analysis 10 independent *E. coli* clones identified *L. infantum* WT and *L. major* MF80.3 alleles in similar proportions indicating that the *L. infantum* hybrid is heterozygous at its *MT* locus. The asterisk (*) indicates the deletion of three nucleotides (M547del) present in *L. major* Friedlin MF80.3 [19] and sequence in red are polymorphisms between *L. major* and *L. infantum*. (D) *L. infantum* 263 WT parasites (Δ) and *L. infantum* 263 hybrid for LmMF80.3Δ*LmjF13.1540*::NEO/*LmjF13.1540* at their *MT* locus (▴) were grown in increasing concentrations of miltefosine and their EC_50_ values determined. The mean of three independent experiments is indicated. A statistical significance was observed by Student's t-test (p<0.05).

The described procedure could prove very useful for applications regarding the role of genomic loci or SNPs in conferring a particular phenotypic trait like virulence or drug resistance. In *Leishmania*, the miltefosine transporter (MT) is a phospholipid flippase located at the plasma membrane of the parasite [42]. Point mutations in MT are correlated to resistance to MF [19], [42], a drug used for the treatment of antimonial-resistant infections in endemic regions [1]. We previously showed that the *L. major* Friedlin LmjF-MF80.3 miltefosine resistant mutant has a three nucleotide deletion (M547del) on both alleles of its *MT* gene (LmjF13.1530) [19]. The inactivating role of the mutations was inferred from transfection of episomal copies of the *MT* gene. In order to reconstruct MF resistance by WGT of LmjF-MF80.3 gDNA, one allele of the *LmjF13.1540* gene located immediately downstream of *MT* on chromosome 13 was replaced by the *NEO* marker in LmjF-MF80.3, giving rise to the LmMF80.3ΔLmjF13.1540::NEO/LmjF13.1540 mutant (Figure S1C). The *LmjF13.1540* gene codes for a protein of unknown function and its inactivation did not alter the MF resistance levels of LmjF-MF80.3 (data not shown). The transfection of *L. infantum* 263 WT parasites with gDNA derived from LmMF80.3ΔLmjF13.1540::NEO/LmjF13.1540 yielded paromomycin-resistant recombinants that integrated the *NEO* marker on the same chromosome as the *MT* gene (chromosome 13) (Figure 5A). Multi-locus PCR sequencing of the *L. infantum* recombinant (Table S11) estimated the size of the exchanged DNA to approximately 25 kb (Figure 5B). Furthermore, degenerate primers allowing the amplification of *MT* from both *L. major* (*LmjF13.1530*) and *L. infantum* (*LinJ13_V3.1590*) were used to amplify the *MT* locus in one representative *L. infantum* hybrid and the cloning of these amplified *MT* fragments into the pGEM-T-easy plasmid revealed a allele frequency of 60/40% for *L. infantum* WT/LmjF-MF80.3 among *E. coli* clones (Figure 5C). The *L. infantum* 263 hybrid thus integrated the M547del mutation from LmjF-MF80.3 on one of its *MT* allele while maintaining the other allele unaltered. This was confirmed by paired-ends next generation sequencing of the whole genome of this hybrid parasite, which further revealed that no other genomic fragment from LmMF80.3ΔLmjF13.1540::NEO/LmjF13.1540 integrated elsewhere in the genome. Whole genome sequencing revealed a single stretch of 29,2 kb with 541 SNPs (301 within coding sequences) derived from *L. major* that spanned positions 596,277 to 625,504 (19 SNPs/kb) on one allele of chromosome 13 that were transferred to *L. infantum* 263. In contrast only 35 SNPs were detected for the rest of the 645 kb sequence of chromosome 13 upstream and downstream the integrated *L. major* DNA fragment (0.05 SNP/kb).

Most importantly, and as a proof-of-principle of our facile strategy to introduce knock-ins in *Leishmania*, an increased resistance to MF was specifically observed for the *L. infantum* hybrid that acquired the M547del from LmjF-MF80.3 at their *MT* locus (Figure 5D).

## Discussion

Homologous gene targeting is a powerful reverse genetic approach allowing to test the functions of specific gene products in trypanosomatid parasites like *Leishmania* [14]. However, available tools only allow performing studies at the level of genes or small DNA fragments and do not allow investigating the role of large genomic loci or of easy assessment of specific point mutations in a natural genomic context. The most critical parameters for successful homologous recombination in *Leishmania* are the degree of homology and the length of homologous sequences between the donor and recipient DNAs [15]. While gene content and synteny is highly conserved between species of *Leishmania*, the high degree of variations observed at the level of nucleotide sequences [2], [3] usually preclude the use of unique DNA constructs for targeting homologous loci between species (Figures 4D, 4F). Genetic exchange among natural populations of *Leishmania* has long been suspected [10], [43] and recently received experimental confirmation [11], with some hybrids even reported to acquire increased fitness or transmission potential [44]. In rare cases are these hybrids crossing the species barrier but natural hybrids between *L. major* and *L. infantum* have been described [10].

In this study, we describe a protocol based on WGT that enables the transfer of large DNA fragments between strains and species of *Leishmania*. Integrations occurred at different genomic loci on distinct chromosomes in recipient cells and up to 45 kb of heterologous gDNA was exchanged between species. Such size limitation could be due to several factors like breakage of the high molecular weight DNA during transformation or structural variations in the genome of recipient cells that would disrupt the progress of recombination. This contrasts with the genome-wide heterozygosity that seems to be happening for hybrids generated in the sand fly vector either in a natural context [10] or in experimental settings [11]–[13]. Notwithstanding, genetic crosses performed in the sand fly vector normally only delimits phenotypic traits to loci covering tens of genes and our approach should thus reveal a valuable complement for narrowing down the list of candidates. Targeted sequencing analyses indicated that recombination events took place between and within orthologous genes in hybrid recombinants, which is consistent with the extensive synteny of *Leishmania* genomes [2]. Interestingly, we noticed that in the two events where the whole genomes of hybrids were sequenced those recombination events occurred in regions of highest homology (recombination sites were in regions of 95% identities while the average region was 90% identical).

The presence of a selection marker on either one or two alleles in the donor gDNAs did not affect the efficiency of transformation (results not shown) but the rates of targeting were different depending on the donor and recipient strains of *Leishmania*. Indeed, our results are consistent with the degree of divergence at the nucleotide level between *Leishmania* species [2], [45] since we were unable to obtain *L. major* or *L. infantum* hybrids when using donor gDNAs derived from two independent *L. (V.) braziliensis* lines having a *NEO* marker integrated at distinct genomic locations,(result not shown). This correlates with the recombination events described in natural populations of *Leishmania*, which mainly implicates closely related species like *L. major/L. infantum* [10], *L. panamensis/L. braziliensis* or *L. panamensis/L. guyanensis* [46]–[48]. We focused our transformation experiments with *L. infantum* as the recipient cell of heterologous DNA. We succeeded once in creating a *L. major hybrid with L. infantum* DNA (results not shown) but this was more difficult and transformation of other *L. infantum* gDNA donors in *L. infantum* never led to *L. major* transformants being either integrated or episomal (data not shown). This may relate to the ten-fold lower efficiency of transformation of *L. major* [49], [50] and one might thus be successful at obtaining *L. major* recombinant hybrids by improving transfection efficiencies.

While we were able to recover recombinants for most of the genomic loci tested, some chromosomal locations were nonetheless targeted less efficiently. The MMR machinery plays a critical role in maintaining genetic stability by correcting for base mismatches that can arise through replication errors or chemical damage [51] and also influences the frequency of homologous recombination between divergent sequences [41], [52]. Interestingly, interfering with the MMR machinery in *Leishmania* increased the number of hybrid clones for these loci less amenable to hybrid formation, probably by dimming the barriers for recombination between mismatched DNA (Figure 4). We also found a background of transfectants maintaining the selection marker as part of extrachromosomal amplicons for most *loci* (Figure 2A, lane 4; Figure 3B, lane 2, Table 1). The hybridization of chromosome-sized blots from several independent hybrid clones indicated that the relative abundance of chromosomal targeting and episomal maintenance of the selection marker varied depending on the donor gDNAs but in the majority of clones, the DNA was integrated (Table 1). Extrachromosomal circles can be generated by homologous recombination between repeated genomic sequences in *Leishmania* [53], [54]. It is thus likely that these circles were generated by recombination between repeated sequences present on large DNA fragments including the *NEO* marker. Extrachromosomal circles were not stable and were lost in the absence of selective pressure (not shown).

Whole genome transformation in naturally competent bacteria was shown to lead to the acquisition of several distinct donor DNA segments that optimally replace up to 3% of the genome of recipient cells [35]. This is in contrast to the unique integration events observed in the genome of *Leishmania* hybrids (Figure 2B, Figure 5B), for which homologous recombination were restricted to genomic loci surrounding the selection marker as shown by whole genome sequencing. It may be possible to further increase the efficiency of recombination by manipulating the expression of recombination enzymes and more loci could be targeted by the use of additional selection markers in the same cell. On the other hand, this controlled recombination prevents the likelihood of phenotypic artefacts due to surrogate DNA exchange events. The method presented here is now allowing the *in vitro* generation of hybrid parasites allowing for testing for gene functions in a natural genomic context. This technique of hybrid formation has also the potential to be useful for other microbial pathogens.

## Supporting Information

Figure S1 **Targeted replacement of the** ***L. major*** **Friedlin genes.** SKO parasites for the genes *LmjF01.0315* and *LmjF05.0610* were generated in *L. major* Friedlin WT parasites (A and B respectively) while the gene LmjF13.1540 was inactivated by NEO in the mutant MF80.3 of *L. major* Friedlin parasites (C). (A) Schematic drawing of the *LmjF01.0315* locus with *Sac*I sites of *L. major* and the respective Southern blot analysis hybridized with a 5′ UTR probe (a ∼500 bp fragment just downstream the start codon of the gene). (B) Schematic drawing of the *LmjF05.0610* locus with *Sac*I sites of *L. major* and the respective Southern blot analysis hybridized with a 5′ UTR probe (a ∼500 bp fragment just upstream the start codon of the gene) *L. major* Friedlin (wild-type) (1) and its respective SKO:NEO (2). (C) Schematic drawing of the *LmjF13.1540* locus with *Pst*I sites of *L. major* MF80.3 and the respective Southern blot analysis hybridized with a 5′ UTR probe (a ∼500 bp fragment just upstream the start codon of the gene). *L. major* Friedlin MF80.3 (1) and its respective SKO:NEO (2).
(TIF)

Figure S2 ***MSH2*** **knockout cells grow similarly to wild-type cells but have increased alkylation tolerance.** (A) Growth of promastigotes *in vitro*. Parasites were inoculated at 2×105 cells/ml and then they were counted every 24 hours. The mean of three independent experiments are indicated. *L. infantum* 263 wild-type parasite (Δ), double replacement clone (Li263ΔMSH2::HYG:BLA) (•) and double replacement clone complemented with *MSH2* gene (Li263ΔMSH2::HYG:BLA) [pSP72αPURα-*MSH2*] (▪). (B) Promastigotes parasites were grown in increased concentrations of MNNG (Nmethyl-N′-nitro-N-nitrosoniguanidine) and the EC_50_ values were determined after 72 hours of growth. The mean of three independent experiments are indicated with a statistical significance observed by Student's t-test (p<0.05) (*).
(TIF)

Table S1 **Primers used for generation of** ***Leishmania*** **genes Knockout and cloning of** ***MSH2*** **gene.**
(DOC)

Table S2 **Loci analyzed by multilocus sequencing typing.** Primers forward and reverse were used for both DNA amplification and sequencing.
(DOC)

Table S3 **Loci analyzed by multilocus sequencing typing genes.** Primers forward and reverse were used for both DNA amplification and sequencing (Table S2). The natural polymorphisms between *L. major* and *L. infantum* enabled mapping the size of exchanged DNA by sequencing. The SNPs found in the hybrid 263 (1) of Figure 2 are listed by their respective position in the gene.
(DOC)

Table S4 **Loci analyzed by multilocus sequencing typing genes.** Primers forward and reverse were used for both DNA amplification and sequencing (Table S2). The natural polymorphisms between *L. major* and *L. infantum* enabled mapping the size of exchanged DNA by sequencing. The SNPs found in the hybrid JPCM5 (1) of Figure 2 are listed by their respective position in the gene.
(DOC)

Table S5 **Loci analyzed by multilocus sequencing typing genes.** Primers forward and reverse were used for both DNA amplification and sequencing (Table S2). The natural polymorphisms between *L. major* and *L. infantum* enabled mapping the size of exchanged DNA by sequencing. The SNPs found in the hybrid JPCM5 (2) of Figure 2 are listed by their respective position in the gene.
(DOC)

Table S6 **Loci analyzed by multilocus sequencing typing genes.** Primers forward and reverse were used for both DNA amplification and sequencing (Table S2). The natural polymorphisms between *L. major* and *L. infantum* enabled mapping the size of exchanged DNA by sequencing. The SNPs found in the hybrid *L. infantum* 263 of Figure 3D are listed by their respective position in the gene.
(DOC)

Table S7 **Loci analyzed by multilocus sequencing typing genes.** Primers forward and reverse were used for both DNA amplification and sequencing (Table S2). The natural polymorphisms between *L. major* and *L. infantum* enabled mapping the size of exchanged DNA by sequencing. The SNPs found in the hybrid *L. infantum* JPCM5 of Figure 3D are listed by their respective position in the gene.
(DOC)

Table S8 **Loci analyzed by multilocus sequencing typing genes.** Primers forward and reverse were used for both DNA amplification and sequencing (Table S2). The natural polymorphisms between *L. major* and *L. infantum* enabled mapping the size of exchanged DNA by sequencing. The SNPs found in the hybrid *L. infantum* 263 of Figure 3E are listed by their respective position in the gene.
(DOC)

Table S9 **Loci analyzed by multilocus sequencing typing genes.** Primers forward and reverse were used for both DNA amplification and sequencing (Table S2). The natural polymorphisms between *L. major* and *L. infantum* enabled mapping the size of exchanged DNA by sequencing. The SNPs found in the hybrid 263 (2) of Figure 3F are listed by their respective position in the gene.
(DOC)

Table S10 **Loci analyzed by multilocus sequencing typing genes.** Primers forward and reverse were used for both DNA amplification and sequencing (Table S2). The natural polymorphisms between *L. major* and *L. infantum* enabled mapping the size of exchanged DNA by sequencing. The SNPs found in the hybrid 263 (3) of Figure 3F are listed by their respective position in the gene.
(DOC)

Table S11 **Loci analyzed by multilocus sequencing typing genes.** Primers forward and reverse were used for both DNA amplification and sequencing (Table S2). The natural polymorphisms between *L. major* and *L. infantum* enabled mapping the size of exchanged DNA by sequencing. The SNPs found in the hybrid *L. infantum* 263 of Figure 5 are listed by their respective position in the gene.
(DOC)
