# Supplementary figures and images for: Generation of Leishmania Hybrids by Whole Genomic DNA Transformation
Source: PLoS Negl Trop Dis. 2012 Sep 20;6(9):e1817. doi: 10.1371/journal.pntd.0001817 (PMC3447969; doi:10.1371/journal.pntd.0001817)

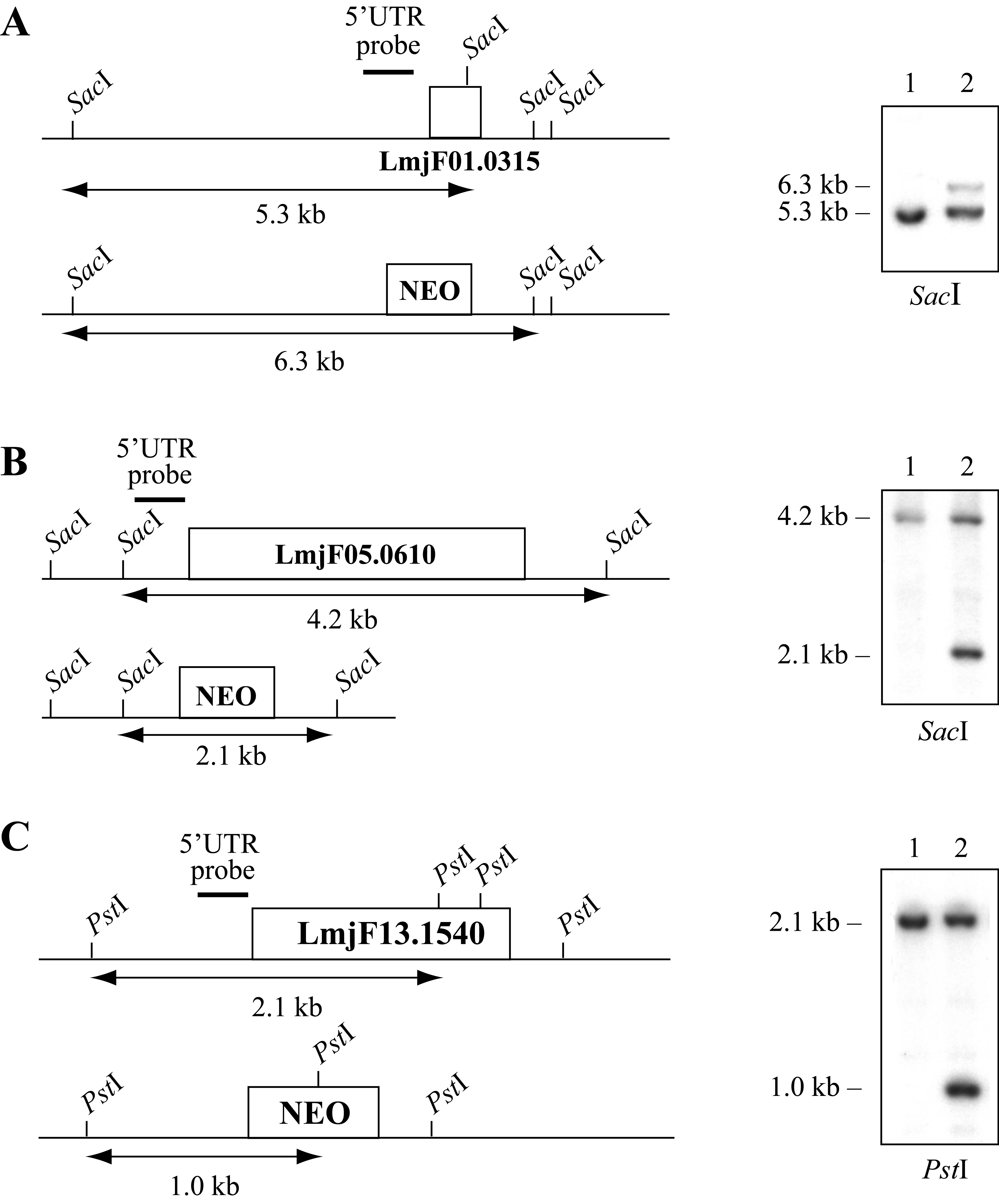

Supplement: Figure S1 — Targeted replacement of the L. major Friedlin genes. SKO parasites for the genes LmjF01.0315 and LmjF05.0610 were generated in L. major Friedlin WT parasites (A and B respectively) while the gene LmjF13.1540 was inactivated by NEO in the mutant MF80.3 of L. major Friedlin parasites (C). (A) Schematic drawing of the LmjF01.0315 locus with SacI sites of L. major and the respective Southern blot analysis hybridized with a 5′ UTR probe (a ∼500 bp fragment just downstream the start codon of the gene). (B) Schematic drawing of the LmjF05.0610 locus with SacI sites of L. major and the respective Southern blot analysis hybridized with a 5′ UTR probe (a ∼500 bp fragment just upstream the start codon of the gene) L. major Friedlin (wild-type) (1) and its respective SKO:NEO (2). (C) Schematic drawing of the LmjF13.1540 locus with PstI sites of L. major MF80.3 and the respective Southern blot analysis hybridized with a 5′ UTR probe (a ∼500 bp fragment just upstream the start codon of the gene). L. major Friedlin MF80.3 (1) and its respective SKO:NEO (2). (TIF) [file pntd.0001817.s001.tar]

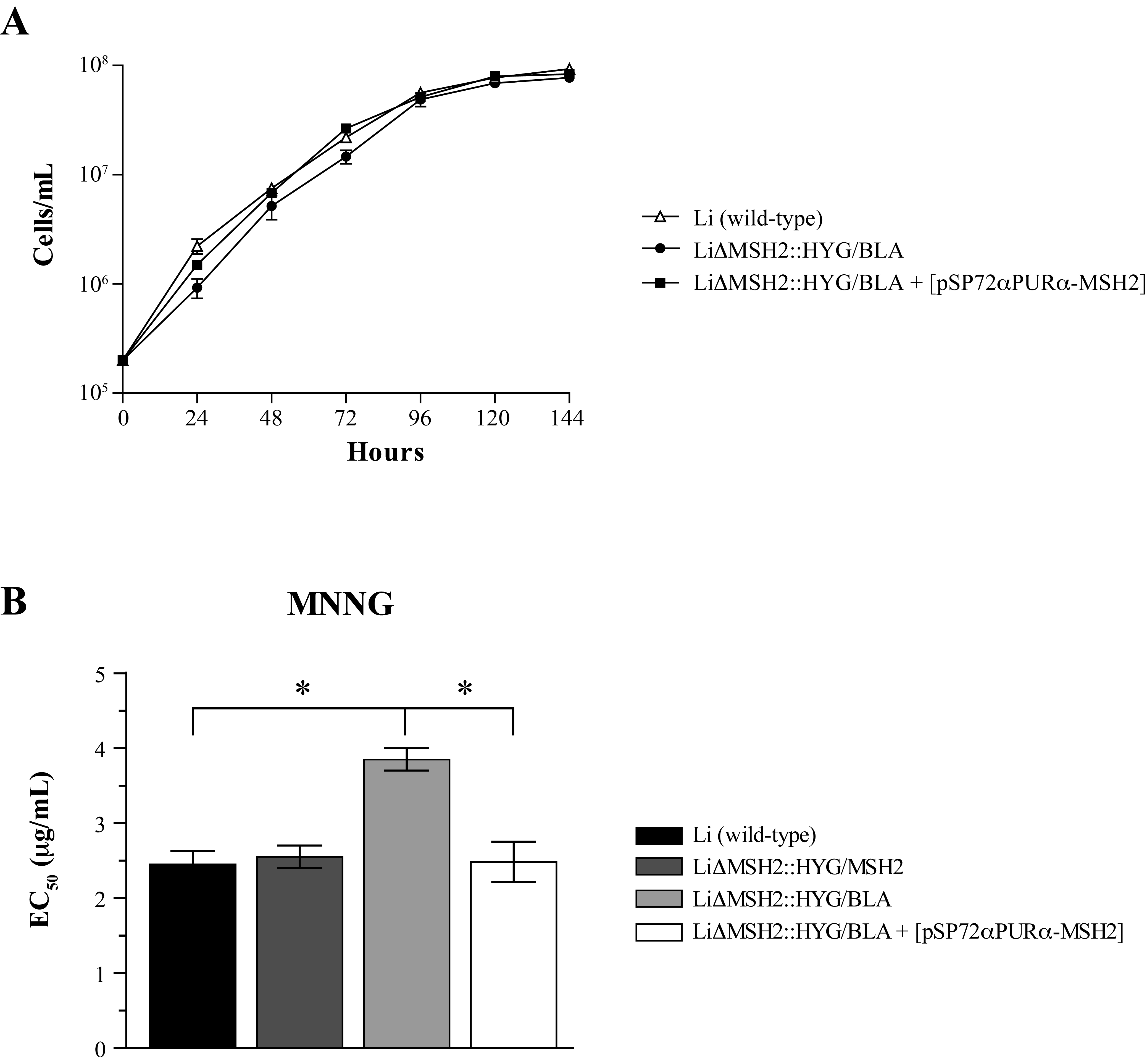

Supplement: Figure S2 — MSH2 knockout cells grow similarly to wild-type cells but have increased alkylation tolerance. (A) Growth of promastigotes in vitro. Parasites were inoculated at 2×10^5^ cells/ml and then they were counted every 24 hours. The mean of three independent experiments are indicated. L. infantum 263 wild-type parasite (Δ), double replacement clone (Li263ΔMSH2::HYG:BLA) (•) and double replacement clone complemented with MSH2 gene (Li263ΔMSH2::HYG:BLA) [pSP72αPURα-MSH2] (▪). (B) Promastigotes parasites were grown in increased concentrations of MNNG (Nmethyl-N′-nitro-N-nitrosoniguanidine) and the EC50 values were determined after 72 hours of growth. The mean of three independent experiments are indicated with a statistical significance observed by Student's t-test (p<0.05) (*). (TIF) [file pntd.0001817.s002.tif]
