# Supplementary material for: Generation of Leishmania Hybrids by Whole Genomic DNA Transformation
Source: PLoS Negl Trop Dis. 2012 Sep 20;6(9):e1817. doi: 10.1371/journal.pntd.0001817 (PMC3447969; doi:10.1371/journal.pntd.0001817)
Supplement: Table S1 — Primers used for generation of Leishmania genes Knockout and cloning of MSH2 gene. (DOC) [file pntd.0001817.s003.doc]

**Table S1.** Primers used for generation of *Leishmania* genes Knockout and cloning of *MSH2* gene.

| **Gene** | **Primers** |
| --- | --- |
| LmjF34.0550 / | A) 5’- CATGCGGGGTGCGTTCTTCTC |
| LinJ34_V3.0570 | B-NEO) 5’- CAATCCATCTTGTTCAATGGCCGATCCCATTTTGTTCACTCTTATTTACA |
| (NEO) | C-NEO) 5’- ATGGGATCGGCCATTGAACA |
|  | D-NEO) 5’-TTTCGTTCGTTGGCCATTGAAACACGTACGATCTTCTCAGAAGAACTCGTCAAGAAGGCG |
|  | E) 5’- GAGGGCTTCGCCAAGGAACAG |
|  | F) 5’- CGCATGTGGGAAAGGCAAAG |
| LmjF13.1540 | A) 5’- CATGTAGCCGGCGCTGTGTG |
| (NEO) | B-NEO) 5’- ACCTGCGTGCAATCCATCTTGTTCAATCATCCGGAACCTAGTGCAGCCGA |
|  | C-NEO) 5’- ATGGGATCGGCCATTGAACA |
|  | D-NEO) 5’- CACCCTCCTCCTCCCCACAGTCAGAAGAACTCGTCAAGAA |
|  | E-NEO) 5’- TCGCCTTCTTGACGAGTTCTTCTGACTGTGGGGAGGAGGAGGGTG |
|  | F) 5’- CGGAAAACAGGGGGTGGCGA |
| (HYG) | B-HYG) 5’- GACGTCGCGGTGAGTTCAGGCTTTTTCATCCGGAACCTAGTGCAGCCGA |
|  | C-HYG) 5’- ATGAAAAAGCCTGAACTCAC |
|  | D-HYG) 5’- GACGTCGCGGTGAGTTCAGGCTTTTTCATCCGGAACCTAGTGCAGCCGA |
|  | E-HYG) 5’- AGCACTCGTCCGAGGGCAAAGGAATAGCTGTGGGGAGGAGGAGGGTG |
| LmjF01.0315 | A) 5’- GTCAGCTTCTACCCGCGACAGT |
| (NEO) | B-NEO) 5’- ACCTGCGTGCAATCCATCTTGTTCAATCATAGCCCCTTGTGAGTGGTGACG |
|  | C-NEO) 5’- ATGGGATCGGCCATTGAACA |
|  | D-NEO) 5’- TCCCTGCACCGCTTAGCCAGTCAGAAGAACTCGTCAAGAA |
|  | E-NEO) 5’- TCGCCTTCTTGACGAGTTCTTCTGACTGGCTAAGCGGTGCAGGGA |
|  | F) 5’- CGTCACATCGTTGCGCAGGC |
| LmjF13.1520 | A) 5’- GCCCCGCAGTCTCGTCAGTT |
| (HYG) | B-HYG) 5’- GACGTCGCGGTGAGTTCAGGCTTTTTCATCCCCCGTGTGTCCTTCACCA |
|  | C-HYG) 5’- ATGAAAAAGCCTGAACTCAC |
|  | D-HYG) 5’- CACCCCCACACCACCACTGTCTATTCCTTTGCCCTCGGAC |
|  | E-HYG) 5’- AGCACTCGTCCGAGGGCAAAGGAATAGACAGTGGTGGTGTGGGGGTG |
|  | F) 5’- CTCATAACGGGGGAGGGGGC |
| LinJ33_V3.0420 (*MSH2*) | A) 5’- CCTGGAGCAGGACGCTGAGT |
|  | B-HYG) 5’- GACGTCGCGGTGAGTTCAGGCTTTTTCATTGCAAGCGTGGTGGAGCACA |
|  | C-HYG) 5’- ATGAAAAAGCCTGAACTCAC |
|  | D-HYG) 5’- CACCACCGCTGAGAACCCCTCTATTCCTTTGCCCTCGGAC |
|  | E-HYG) 5’- AGCACTCGTCCGAGGGCAAAGGAATAGAGGGGTTCTCAGCGGTGGTG |
|  | F) 5’- GCACTGCACACGACTGCCAC |
|  | B-NEO) 5’- ACCTGCGTGCAATCCATCTTGTTCAATCATTGCAAGCGTGGTGGAGCACA |
|  | C-NEO) 5’- ATGGGATCGGCCATTGAACA |
|  | D-NEO) 5’- CACCACCGCTGAGAACCCCTTCAGAAGAACTCGTCAAGAA |
|  | E-NEO) 5’- TCGCCTTCTTGACGAGTTCTTCTGAAGGGGTTCTCAGCGGTGGTG |
|  | B-BLA) 5’- ATTCTTCTTGAGACAAAGGCTTGGCCAT TGCAAGCGTGGTGGAGCACA |
|  | C-BLA) 5’- ATGGCCAAGCCTTTGTCTCA |
|  | D-BLA) 5’- CACCACCGCTGAGAACCCCTTTAGCCCTCCCACACATAAC |
|  | E-BLA) 5’- CTGCCCTCTGGTTATGTGTGGGAGGGCTAAAGGGGTTCTCAGCGGTGGTG |
| *MSH2* probe | 5’ - ACCTACACAGGCCCGCAGTC |
| (554 bp) | 5’ - AGAGCCCAGGCCACGAAGAC |
| *MSH2* (full gene *Xba*I sites) | 5’ - CGC*TCTAGA*CGCACATGCACCTACGCACG |
| (3.5 kb) | 5’ - CGC*TCTAGA*CAAACAAGGATAGCGAGAAG |
