# Supplementary material for: Generation of Leishmania Hybrids by Whole Genomic DNA Transformation
Source: PLoS Negl Trop Dis. 2012 Sep 20;6(9):e1817. doi: 10.1371/journal.pntd.0001817 (PMC3447969; doi:10.1371/journal.pntd.0001817)
Supplement: Table S2 — Loci analyzed by multilocus sequencing typing. Primers forward and reverse were used for both DNA amplification and sequencing. (DOC) [file pntd.0001817.s004.doc]

**Table S2.** Loci analyzed by multilocus sequencing typing. Primers forward and reverse were used for both DNA amplification and sequencing.

| **Chromosome** | **Gene *L. major* / *L. infantum*** | **Primers** | **Fragment size (bp)** |
| --- | --- | --- | --- |
| 34 | LmjF34.0540 / LinJ34_V3.0560 | 5’- GCCGAGGCAGAGGGCTACAT and 5’- TCTCCAGCCCGACCTTTGCG | 771 |
| 34 | LmjF34.0520 / LinJ34_V3.0540 | 5’- GAGCATGACCGGGCTTGGGCT and 5’- CACCGGACGCTTTGTCGTGC | 408 |
| 34 | LmjF34.0560 / LinJ34_V3.0580 | 5’- CGTCACCGTTGCTGCCATCG and 5’- CTTTTACGCCTGCTCGCGCC | 574 |
| 34 | LmjF34.0510 / LinJ34_V3.0530 | 5’- GACGCGGCTCAGACCACAGA and 5’- GGCGAAGCCGCTACACTCCT | 596 |
| 34 | LmjF34.0570 / LinJ34_V3.0590 | 5’- CACTGCCGACGCGAAAGCAC and 5’- GCGCAGCGCTGTTGATCTCG | 575 |
| 34 | LmjF34.0530 / LinJ34_V3.0550 | 5’- CGAGGCGCATCAAGCCCAAC and 5’- TCTCCTGTGCTTCCTGGGGC | 544 |
| 34 | LmjF34.0590 / LinJ34_V3.0610 | 5’- GCTGGCGGTGTCGTCTACCA and 5’- GCATGTGCACCCTCGCCTTG | 565 |
| 34 | LmjF34.0600/ LinJ34_V3.0620 | 5’- GTCGCCTGCAGCAGCACAAG and 5’- GCGGCGGCAGAGTGAGAGTA | 570 |
| 34 | LmjF34.0610/ LinJ34_V3.0630 | 5’- AACGCCGTCGTAGCTGAGGG and 5’- GCGCTCTACCACCTCCGCA | 507 |
| 34 | LmjF34.0080 / [LinJ34_V3.0080](http://old.genedb.org/genedb/Search?organism=linfantum&name=LinJ34_V3.0080) | 5’- ATGTCGGAAGAGCAGTCT and 5’- TCACAGCTTATTCGAGGGAA | 1689 |
| 30 | LmjF30.1230 / LinJ30_V3.1290 | 5’- GAGGAGGACGCGCGGAAAGA and 5’- CCTTTGGTCGCTGCGCCTTG | 522 |
| 30 | LmjF30.1240 / LinJ30_V3.1300 | 5’- GGTGCCGGTTCAGCTGGAGA and 5’- CGGTGTACGGCACGAGTCCA | 598 |
| 30 | LmjF30.1255 / LinJ30_V3.1320 | 5’- GCGTGACGGGCATCTTGCAC and 5’- GACTTGGCCGTTGTGCGGTG | 574 |
| 30 | LmjF30.1260 / LinJ30_V3.1330 | 5’- AGATGCCGGGCCATCCTCTG and 5’- GCGCAGCATCACTTCCAGGC | 540 |
| 30 | LmjF30.1205 / LinJ30_V3.1270 | 5’- GGTGGTGCACCGCTTCATGC and 5’- GCCGACGTTGTCGCTACGC | 199 |
| 30 | LmjF30.1270 / LinJ30_V3.1240 | 5’- CTACCGCAACCTCCTACTT and 5’- CAACTCTCCACTTGCATCAT | 216 |
| 1 | LmjF01.0310 / LinJ01_V3.0310 | 5’-TCCCACGCTGCTAACGGCTC and 5’- CCCCTCGGCAAGGAATTCGG | 619 |
| 1 | LmjF01.0290 / LinJ01_V3.0290 | 5’- AGATCAGATCGGCTATGATGAGACGG and 5’- GGGTGCGTAGTCGTGCTTCCA | 412 |
| 1 | LmjF01.0280 / LinJ01_V3.0280 | 5’- CGAGGAGCTGTCCCGCAAGT and 5’- CCCGCTCGTCTCGGTGTCAA | 535 |
| 1 | LmjF01.0320 / LinJ01_V3.0330 | 5’- ATCACCGGAAGCTGGGACGG and 5’- ACCACTCTTCCCCATGGCCC | 591 |
| 1 | LmjF01.0340 / LinJ01_V3.0360 | 5’- GCCAGACGCCGCTACAAAGC and 5’- AGACGACGGGTCAAGCACCG | 522 |
| 1 | LmjF01.0240 / LinJ01_V3.0240 | 5’- GCAGGTGGCCGCCTTTCTTG and 5’- GATGGCGGCCTGCAACTTCG | 568 |
| 1 | LmjF01.0260/ LinJ01_V3.0260 | 5’- TGAGCAGGAGGCGCTCAACC and 5’- CCTTCTCTGCCGCCAGCTCT | 533 |
| 1 | LmjF01.0360 / LinJ01_V3.0380 | 5’- CGAAGCGCGTGCCCTCTCTA and 5’- TCGCTGCTGCCGTAGACCAG | 445 |
| 1 | LmjF01.0380 / LinJ01_V3.0400 | 5’- ACGAGCTGCTCTACGTCCGC and 5’- GGCCAGCGCTCAACAACGTC | 531 |
| 1 | LmjF01.0210 / LinJ01_V3.0210 | 5’- GCGGATTCTGGAACGGCAGC and 5’- GCCACCGTCGCACTCACCAT | 535 |
| 1 | LmjF01.0230 / LinJ01_V3.0230 | 5’- TGGGTGGCCTGAGCATCACC and 5’- CGGAGAAGTTGCGAAGCCGC | 360 |
| 1 | LmjF01.0390 / LinJ01_V3.0410 | 5’- TCATGAGCAAGCCGCAGGCA and 5’- ATGGCGTCGGCGAAGAAGCA | 559 |
| 1 | LmjF01.0410 / LinJ01_V3.0430 | 5’- GCGACCTCGAGGAGAGCGTC and 5’- ACCTGCTGCAGCTTCGGGTT | 541 |
| 13 | LmjF13.1530 / LinJ13_V3.1590 | 5’- AAGCTAGGCGACGCTCTCGG and 5’- GAGCTGCGGTTGTGCAGGGA | 600 |
| 13 | LmjF13.1520 / LinJ13_V3.1580 | 5’- CAGGAGCGGGCGTACGTCTT and 5’- CTCCGCCTGGTTCACTCGCT | 531 |
| 13 | LmjF13.1500 / LinJ13_V3.1550 | 5’- CGTTTCTCGGGAGCGCCGTA and 5’- CGGCTCGGAGTCGAGGAGAG | 557 |
| 13 | LmjF13.1550 / LinJ13_V3.1610 | 5’- GCCCTTGTCGACGTGCTTGC and 5’- GGCGGTTGCGTTCTCTGTGC | 553 |
| 13 | LmjF13.1570 / LinJ13_V3.1630 | 5’- CCCCACATCCAGGGCATCCA and 5’- TGCGTGTACTCGTCGCCGTC | 575 |
| 13 | LmjF13.1540 / LinJ13_V3.1600 | 5’- CACTTTCACGATGCCCGCCC and 5’- CGCGCGTTTCTACGCTGCTG | 546 |
