# Supplementary material for: Generation of Leishmania Hybrids by Whole Genomic DNA Transformation
Source: PLoS Negl Trop Dis. 2012 Sep 20;6(9):e1817. doi: 10.1371/journal.pntd.0001817 (PMC3447969; doi:10.1371/journal.pntd.0001817)
Supplement: Table S3 — Loci analyzed by multilocus sequencing typing genes. Primers forward and reverse were used for both DNA amplification and sequencing (Table S2). The natural polymorphisms between L. major and L. infantum enabled mapping the size of exchanged DNA by sequencing. The SNPs found in the hybrid 263 (1) of Figure 2 are listed by their respective position in the gene. (DOC) [file pntd.0001817.s005.doc]

**Table S3.** Loci analyzed by multilocus sequencing typing genes. Primers forward and reverse were used for both DNA amplification and sequencing (Table S2). The natural polymorphisms between *L. major* and *L. infantum* enabled mapping the size of exchanged DNA by sequencing. The SNPs found in the hybrid 263 (1) of Figure 2 are listed by their respective position in the gene.

| **Gene / SNPs positions** | ***L. major* Friedlin** | | ***L. infatum* JPCM5** | **Hybrid *L. infantum* 263 (1)** |
| --- | --- | --- | --- | --- |
| LmjF34.0510 / LinJ34_V3.0530 (596 bp fragment) | | | | |
| 1063 | A | | C | C |
| 1156 | T | | C | C |
| 1161 | C | | T | T |
| 1180 | A | | T | T |
| 1190 | A | | G | G |
| 1222 | T | | G | G |
| 1241 | G | | A | A |
| 1260 | T | | C | C |
| 1283 | A | | G | G |
| 1286 | A | | G | G |
| 1326 | T | | C | C |
| 1329 | C | | T | T |
| 1344 | A | | C | C |
| 1359 | A | | G | G |
| 1392 | A | | T | T |
| 1411 | A | | G | G |
| 1418 | C | | A | A |
| 1446 | G | | A | A |
| 1456 | C | | T | T |
| 1468 | C | | G | G |
| 1472 | A | | G | G |
| LmjF34.0520 / LinJ34_V3.0540 (408 bp fragment) | | | | |
| 3696 | A | | G | A/G |
| 3705 | G | | A | G/A |
| 3717 | A | | G | A/G |
| 3733 | A | | C | A/C |
| 3742 | A | | C | A/C |
| 3748 | A | | C | A/C |
| 3749 | C | | A | C/A |
| 3750 | G | | T | G/T |
| 3756 | T | | C | T/C |
| 3767 | T | | C | T/C |
| 3768 | C | | G | C/G |
| 3783 | C | | G | C/G |
| 3818 | C | | T | C/T |
| 3839 | T | | C | T/C |
| 3841 | G | | A | G/A |
| 3844 | G | | A | G/A |
| 3855 | A | | G | A/G |
| 3873 | C | | T | C/T |
| 3877 | A | | G | A/G |
| 3886 | C | | G | C/G |
| 3897 | C | | T | C/T |
| 3916 | G | | A | G/A |
| 3926 | G | | T | G/T |
| 3939 | C | | T | C/T |
| 3940 | A | | G | A/G |
| 3946 | A | | G | A/G |
| 3953 | C | | T | C/T |
| 3987 | T | | C | T/C |
| LmjF34.0530 / LinJ34_V3.0550 (544 bp fragment) | | | | |
| 299 | C | | A | C/A |
| 303 | T | | G | T/G |
| 305 | A | | C | A/C |
| 306 | G | | T | G/T |
| 311 | A | | C | A/C |
| 332 | T | | C | T/C |
| 340 | C | | A | C/A |
| 343 | G | | C | G/C |
| 347 | G | | A | G/A |
| 352 | C | | G | C/G |
| 357 | A | | G | A/G |
| 359 | A | | G | A/G |
| 378 | C | | A | C/A |
| 399 | C | | G | C/G |
| 411 | C | | G | C/G |
| 412 | A | | G | A/G |
| 420 | C | | A | C/A |
| 428 | A | | G | A/G |
| 437 | G | | C | G/C |
| 444 | G | | C | G/C |
| 454 | G | | A | G/A |
| 469 | A | | G | A/G |
| 474 | C | | G | C/G |
| 479 | G | | A | G/A |
| 504 | G | | T | G/T |
| 511 | G | | A | G/A |
| 512 | T | | G | T/G |
| 514 | T | | G | T/G |
| 518 | C | | A | C/A |
| 531 | A | | C | A/C |
| 538 | G | | T | G/T |
| 539 | A | | G | A/G |
| 541 | G | | A | G/A |
| 552 | G | | A | G/A |
| 555 | C | | T | C/T |
| 616 | G | | A | G/A |
| 624 | C | | T | C/T |
| 630 | G | | C | G/C |
| 638 | C | | T | C/T |
| LmjF34.0560 / LinJ34_V3.0580 (574 bp fragment) | | | | |
| 2994 | A | | G | G/A |
| 2997 | C | | A | A/C |
| 3030 | T | | C | C/T |
| 3070 | A | | G | G/A |
| 3072 | C | | T | T/C |
| 3076 | T | | G | G/T |
| 3092 | C | | T | T/C |
| 3109 | T | | G | G/T |
| 3117 | T | | C | C/T |
| 3129 | C | | T | T/C |
| 3130 | G | | T | T/G |
| 3131 | T | | A | A/T |
| 3135 | C | | G | G/C |
| 3152 | C | | A | A/C |
| 3153 | G | | A | A/G |
| 3163 | C | | T | T/C |
| 3169 | T | | G | G/T |
| 3186 | T | | G | G/T |
| 3211 | A | | G | G/A |
| 3231 | G | | C | C/G |
| 3232 | C | | G | G/C |
| 3240 | A | | G | G/A |
| 3253 | T | | C | C/T |
| 3292 | G | | A | A/G |
| 3303 | G | | T | T/G |
| 3345 | A | | G | G/A |
| 3368 | G | | A | A/G |
| 3397 | C | | T | T/C |
| 3415 | C | | A | A/C |
| 3416 | G | | C | C/G |
| LmjF34.0570 / LinJ34_V3.0590 (575 bp fragment) | | | | |
| 1437 | | C | T | T/C |
| 1439 | | A | C | C/A |
| 1449 | | G | A | A/G |
| 1507 | | A | G | G/A |
| 1510 | | G | A | A/G |
| 1602 | | A | G | G/A |
| 1635 | | C | G | G/C |
| 1653 | | A | G | G/A |
| 1665 | | T | G | G/T |
| 1716 | | T | C | C/T |
| 1725 | | G | A | A/G |
| 1758 | | A | G | G/A |
| 1762 | | A | G | G/A |
| 1764 | | A | G | G/A |
| 1818 | | T | C | C/T |
| 1821 | | C | T | T/C |
| 1906 | | A | G | G/A |
| LmjF34.0600 / LinJ34_V3.0620 (570 bp fragment) | | | | |
| 1017 | | T | C | C/T |
| 1032 | | C | T | T/C |
| 1061 | | T | A | A/T |
| 1131 | | C | T | T/C |
| 1137 | | G | A | A/G |
| 1155 | | T | C | C/T |
| 1212 | | A | G | G/A |
| 1228 | | A | G | G/A |
| 1267 | | C | T | T/C |
| 1308 | | T | C | C/T |
| 1314 | | C | T | T/C |
| 1335 | | T | C | C/T |
| 1338 | | A | G | G/A |
| 1368 | | T | C | C/T |
| LmjF34.0610 / LinJ34_V3.0630 (507 bp fragment) | | | | |
| 499 | | A | G | G |
| 540 | | G | A | A |
| 549 | | G | A | A |
| 550 | | A | G | G |
| 686 | | C | A | A |
| 693 | | G | A | A |
| 717 | | T | C | C |
| 769 | | G | A | A |
| 789 | | C | T | T |
| 795 | | C | G | G |
| 807 | | A | G | G |
| 822 | | A | G | G |
| 846 | | C | T | T |
| 861 | | G | A | A |
