# Supplementary material for: Generation of Leishmania Hybrids by Whole Genomic DNA Transformation
Source: PLoS Negl Trop Dis. 2012 Sep 20;6(9):e1817. doi: 10.1371/journal.pntd.0001817 (PMC3447969; doi:10.1371/journal.pntd.0001817)
Supplement: Table S8 — Loci analyzed by multilocus sequencing typing genes. Primers forward and reverse were used for both DNA amplification and sequencing (Table S2). The natural polymorphisms between L. major and L. infantum enabled mapping the size of exchanged DNA by sequencing. The SNPs found in the hybrid L. infantum 263 of Figure 3E are listed by their respective position in the gene. (DOC) [file pntd.0001817.s010.doc]

**Table S8.** Loci analyzed by multilocus sequencing typing genes. Primers forward and reverse were used for both DNA amplification and sequencing (Table S2). The natural polymorphisms between *L. major* and *L. infantum* enabled mapping the size of exchanged DNA by sequencing. The SNPs found in the hybrid *L. infantum* 263 of Figure 3E are listed by their respective position in the gene.

| **Gene / SNPs positions** | ***L. major* Friedlin** | ***L. infatum* JPCM5** | **Hybrid *L. infantum* 263** |
| --- | --- | --- | --- |
| LmjF01.0210 / LinJ01_V3.0210 (535 bp fragment) | | | |
| 193 | T | C | C |
| 211 | G | A | A |
| 213 | T | C | C |
| 225 | T | C | C |
| 246 | G | A | A |
| 270 | G | C | C |
| 273 | C | T | T |
| 278 | T | C | C |
| 290 | C | T | T |
| 312 | A | G | G |
| 318 | G | C | C |
| 342 | C | T | T |
| 351 | C | T | T |
| 357 | T | C | C |
| 366 | C | T | T |
| 396 | T | C | C |
| 436 | G | T | T |
| 462 | C | A | A |
| 467 | C | T | T |
| 469 | A | G | G |
| 471 | C | T | T |
| 531 | C | T | T |
| 536 | T | A | A |
| 539 | T | C | C |
| 586 | G | A | A |
| LmjF01.0230 / LinJ01_V3.0230 (360 bp fragment) | | | |
| 390 | C | T | T |
| 394 | A | G | G |
| 449 | G | T | T |
| 454 | G | T | T |
| 472 | A | C | C |
| 473 | T | C | C |
| 476 | C | T | T |
| 481 | T | C | C |
| 482 | G | A | A |
| 490 | G | A | A |
| 524 | G | A | A |
| 531 | T | C | C |
| 535 | G | T | T |
| 542 | C | T | T |
| 561 | C | G | G |
| 597 | T | C | C |
| 601 | A | G | G |
| LmjF01.0240 / LinJ01_V3.0240 (568 bp fragment) | | | |
| 545 | C | T | C/T |
| 590 | T | A | T/A |
| 664 | G | A | G/A |
| 680 | G | C | G/C |
| 719 | G | A | G/A |
| 787 | T | C | T/C |
| 826 | A | G | A/G |
| 847 | G | A | G/A |
| 859 | G | C | G/C |
| 923 | G | A | G/A |
| 931 | T | C | T/C |
| 935 | A | G | A/G |
| 946 | C | G | C/G |
| LmjF01.0290 / LinJ01_V3.0290 (412 bp fragment) | | | |
| 123 | A | C | A/C |
| 132 | C | T | C/T |
| 186 | T | C | T/C |
| 204 | C | T | C/T |
| 214 | T | C | T/C |
| 303 | G | A | G/A |
| 341 | A | G | A/G |
| 354 | G | A | G/A |
| 359 | C | T | C/T |
| LmjF01.0310 / LinJ01_V3.0310 (619 bp fragment) | | | |
| 606 | T | C | T/C |
| 629 | G | A | G/A |
| 650 | G | C | G/C |
| 656 | A | C | A/C |
| 660 | C | T | C/T |
| 663 | G | A | G/A |
| 675 | A | G | A/G |
| 681 | G | T | G/T |
| 706 | G | A | G/A |
| 721 | C | G | C/G |
| 735 | A | G | A/G |
| 787 | G | A | G/A |
| 801 | T | C | T/C |
| 843 | G | T | G/T |
| 889 | T | C | T/C |
| 904 | G | C | G/C |
| 964 | C | A | C/A |
| 969 | C | T | C/T |
| 978 | C | G | C/G |
| 1005 | C | T | C/T |
| 1023 | T | C | T/C |
| 1027 | G | A | G/A |
| 1080 | C | G | C/G |
| LmjF01.0380 / LinJ01_V3.0400 (531 bp fragment) | | | |
| 1955 | G | A | G/A |
| 2020 | G | A | G/A |
| 2024 | C | G | C/G |
| 2033 | T | C | T/C |
| 2104 | A | G | A/G |
| 2170 | G | A | G/A |
| 2174 | A | C | A/C |
| 2184 | G | A | G/A |
| 2190 | G | A | G/A |
| 2200 | A | G | A/G |
| 2204 | T | C | T/C |
| 2219 | A | G | A/G |
| 2224 | T | C | T/C |
| 2250 | T | A | T/A |
| 2258 | G | AA | G/A |
| 2259 | T | C | T/C |
| 2271 | A | G | A/G |
| 2301 | G | C | G/C |
| 2312 | T | C | T/C |
| 2324 | C | G | C/G |
| 2333 | T | C | T/C |
| LmjF01.0400 / LinJ01_V3.0420 | | | |
| 1012 | C | T | C/T |
| 1104 | G | A | G/A |
| 1114 | T | C | T/C |
| 1145 | T | C | T/C |
| 1157 | C | T | C/T |
| 1178 | A | G | A/G |
| 1180 | C | T | C/T |
| 1205 | G | A | G/A |
| 1232 | G | A | G/A |
| 1238 | A | G | A/G |
| 1247 | G | T | G/T |
| 1285 | T | C | T/C |
| 1294 | G | A | G/A |
| 1295 | A | G | A/G |
| 1300 | C | T | C/T |
| 1305 | C | T | C/T |
| 1307 | G | A | G/A |
| 1313 | A | G | A/G |
| 1334 | T | C | T/C |
| 1341 | G | C | G/C |
| 1373 | G | T | G/T |
| LmjF01.0410 / LinJ01_V3.0430 (541 bp fragment) | | | |
| 144 | T | C | C |
| 237 | C | T | T |
| 249 | C | T | T |
| 261 | A | G | G |
| 275 | G | A | A |
| 301 | C | A | A |
| 381 | C | T | T |
| 493 | A | G | G |
